# Education can modify the long term impact of early childhood famine exposure on adulthood economic achievement: a historical cohort study among the survivors of the great Ethiopian famine 1983–85

**DOI:** 10.1186/s13690-021-00564-w

**Published:** 2021-05-04

**Authors:** Kalkidan Hassen Abate, Misra Abdullahi, Fedlu Abdulhay, Getachew Arage, Mohammed Mecha, Mohammed Yenuss, Habtamu Hassen, Tefera Belachew

**Affiliations:** 1 Department of Nutrition and Dietetics, Institute of Health, Jimma University, Jimma, Ethiopia; 2 Department of Population and Family Heath, Institute of Health, Jimma University, Jimma, Ethiopia; 3 Department of Obstetrics and Gynecology, Faculty of Medical Sciences, Jimma University, Jimma, Ethiopia; 4 Department of Nutrition and Dietetics, College of Health Sciences, DebreTabor University, Debre Tabor, Ethiopia; 5 Department of Internal Medicine, Faculty of Medical Sciences, Jimma University, Jimma, Ethiopia; 6 Department of Environmental Health Science, College of Health and Medical Sciences, Wollo University, Dessie, Ethiopia

**Keywords:** Famine, Wealth, Economic achievement

## Abstract

**Background:**

Previous famine studies reported the inverse link between early life nutritional deprivation and adulthood optimal health outcomes. However, there remain sparse data on the impact of early life famine exposure in later life economic achievement. Hence, we set out to examine the association of early life famine exposure on economic achievement among survivors of the 1983–85 great Ethiopian famine.

**Method:**

A historical cohort study design was employed among 968 adult men and women in the Raya Kobo district, Northern Ethiopia. Participants were categorized into in utero exposed, postnatal exposed and unexposed groups based on self-reported age and birthdate. Structured questionnaire was used to collect data on socio-demographic and individual assets. Principal component analysis (PCA) was used to determine wealth index as proxy for economic achievement. Multinomial logistic regression analyses were employed to examine the independent effect of early life famine exposure on the outcome.

**Results:**

In unadjusted model, compared to unexposed cohorts, in utero and postnatal famine exposed cohorts were nearly twice more likely to fall in the lowest wealth category (OR: 1.93, 95% CI: 1.40, 2.64) and (OR: 2.12, 95%CI: 1.45, 3.08), respectively. However, these associations became non-significant when adjusted for biologic and demographic variables (*P* > 0.05). Instead, educational status appeared to have significant association with wealth; those who can’t read or write among in utero and postnatal exposed group were three times more likely to fall in low wealth index category than those who achieved secondary and above level of education (OR = 3.00 95% CI: 1.74, 5.18) and (OR = 2.92, 95% CI: 1.48, 5.76), respectively. Similarly, those with primary education among in uero and postnatal famine exposed cohorts were twice more likely to fall in the low wealth index than compared to those secondary and above level of education (OR = 2.04 95% CI: (1.18, 3.54) and (OR = 2.17 95% CI: 1.12, 4.22), respectively.

**Conclusion:**

Education appears to be a significant independent factor to determine one’s economic achievement in the studied famine cohort. This may imply, the possible impact of early life famine exposure on economic achievement later in adult life could be modified through better education. Our findings justify the need of expanding education in hunger spots in general and in famine settings in particular.

**Supplementary Information:**

The online version contains supplementary material available at 10.1186/s13690-021-00564-w.

## Background

According to the World Health Organization (WHO), poor nutrition is the single most important threat to the world’s health [1]. There exists sufficient evidence linking food insecurity and or undernutrition with the unfavorable short term health, growth and development outcomes among children [2]. Evidences are also mounting on the link between early life health and nutrition adversaries with long term sequels through the life course and subsequent generations [2–4]. More importantly, nutritional deficiencies from conception to 2 years, i.e. the first 1000 days could have irreversible lifelong health consequences [5].

The link between undernutrition and economic wellbeing appear to be bidirectional [6]. It is obvious that optimal nutritional intake maintains health in turn raises productivity, thereby, brings economic growth [6–8]. Subsequently, the higher economic growth in turn can enhance better nutritional intake [7, 8]. There are ample reports which indicate loss of both human and economic potential due to failing to address undernutrition [6–9]. For example, the UNICEF estimates the average lifetime lost earnings associated with stunting as minimum as 1400 USD per child [10]. These damages are attributed to irreversible structural and functional damages to the body, resulting shorter in stature and delays in the development of cognitive function [6–10].

To investigate the long term impacts of early life undernutrition on adult health and wellbeing, studies have used three approaches in the past; Modeling using instrumental variables (e.g., Alderman et al, 2006 [11], through interventional studies (e.g., Halim, et al., 2015 [12] and using old famines as a natural experiments [13–16]. Evidence from modeling studies were not empirical while interventional studies are expensive and methodologically complicated, thus not often conducted [11, 12]. Conversely, famine cohorts can serve as a natural experimental setting where cohorts’ outcome can be evaluated for childhood exposure. In doing so, understanding the present impact of old famines can give us an opportunity to generate evidence from fetal and first 2 years of postnatal life as a basis of adulthood illness [15, 16]. Furthermore, such endeavor can enhance the capability to predict long term consequences of the present catastrophic events such as floods, storms and droughts which have been doubled since 1990 [4].

In earlier famine studies, there are adequate evidences justifying the inverse link between early life adverse environment and later life suboptimal health outcomes [13, 16–18]. There also some indication on the adversary of prenatal famine on outstanding precursors of economic achievements such as cognitive function [19, 20], educational attainment [21, 22] and height [11, 17–23]. However, there remain sparse data on the impact of early life famine exposure on survivors’ economic achievement, though can be considered as the consequential outcome of the studied biologic, health and cognitive parameters [13–16, 18, 19]. Data from the famine studies in China (1959–61) [24] and Greece (1941–42) [25], Dutch Hunger (1944–45) [26] and the food crisis in Germany (1944–48) [27] reported inconsistent findings. There was also heterogeneity in estimate measures used among these studies as adult monthly wage, lifetime earnings, wealth index and other surrogates were used. Nonetheless, lower reductions in some measures of income and lower occupational status were reported among Chinese and Greek famine survivors, respectively [24, 25]. In contrast, there was no evidence of adverse economic effects of exposure to the German food crisis as well as the Dutch famine survivors were reported [26, 27]. Meanwhile Alderman et al. (2006) reported drought exposure in childhood in Zimbabwe led a 14% reduction in lifetime earnings, but this estimate was calibrated from schooling likely gains [11].

The great Ethiopian famine (1983 to 1985) is one of the recent foes in human history, which left 1.2 million dead, 2.5 million people internally displaced and nearly 200,000 children were orphaned [28, 29]. In our earlier studies in the same setting, we have reported the impacts of the famine on anthropometric, cognitive and metabolic markers among survived adults [30–32]. Those findings clearly indicated the impact of the great famine on adult height, cognitive function and health outcomes which consequently could have an impact on economic achievement. It was a plausible assumption, however, we couldn’t locate any empirical study focusing on the impact of childhood famine on adulthood economic achievement. Thus, we set out to investigate the association between famine exposure to the great famine of Ethiopia in utero and first 2 years of postnatal life (first 1000 days) and economic achievement durig adulthood.

## Method

### Study setting, design and period

The study was conducted in the Raya Kobo district, North Wollo Zone, Ethiopia. The district has a total population of 228,798 of which 147,837 are females and covers an area of 2001.57 km^2^ [33]. A historical cohort study design was employed from March to April, 2019 to investigate the effect of early life famine exposure on economic achievement in adultds.

### Study participants, sample size determination and sampling procedure

Those who conceived or born between August 8, 1983 and August 30, 1985 in the setting were taken as in utero famine exposed groups. Whereas, participants born between September 8, 1981 to August 8, 1983 were considered as postnatal exposed (birth to 2 years during the famine period). The post-famine cohorts (unexposed) were those born between 8 September 1987 and 8 October, 1988. Participants born immediately after end of the famine (between September 8, 1986 to August 30, 1987) were excluded inorder to minimize the misclassification of famine exposure among exposed and unexposed groups (Supplementary file 1). Additionally, exclusions were made for subjects with physical disability, including deformity (Kyphosis, Scoliosis, and limb deformity), pregnancy and history of household displacement during the famine period to other areas of the country.

The sample size was calculated by applying two population proportions using OpenEpi and taking the following assumptions: type one error 5, 80% power, Ratio of sample size, exposed/ unexposed of 1 and effect size of 2% [21]. Hence, the total calculated sample size was 968 (333, 302 and 333 in utero, postnatal and unexposed groups). Multistage stratified random sampling method was used to recruit the participants (Supplementary file 2).

### Measurements

Self-reported birth date and age of the participants were used to classify the status of famine exposure. The outcome measure of the study, adulthood economic achievement was determined by a proxy indicator, wealth index, given the lack of reliable data on income or expenditures in low income countries.

Anthropometric measurements were taken using a standardized protocol [33]. Portable battery operated digital scale and stadiometer (Seca®, Germany) was used to measure weight and height of participants, respectively. Cognitive assessment was measured using the Montreal Cognitive Assessment (MoCA) as we reported in details elsewhere [32]. In addition, data on different sets of sociodemographic covariates were collected using a structured questionnaire.

### Data processing and analysis

Data were entered using EpiData 3.1 and analyzed using SPSS for windows version 25 ([SPSS Inc. version 25, Chicago, Illinois]. Categorical variables were described as frequencies and percentages and compared using the Pearson chi-square test. Continuous variables with a normal distribution were described using the relevant indicators of central tendency and spread (mean ± SD). Continuous variables with skewed distribution were reported as medians ± IQR. Student’s t - test was used to evaluate the mean score difference between famine exposed and non-exposed cohorts. Wealth index was generated using Principal Components Analysis (PCA). The scores for 25 types of assets and utilities were translated into latent factors and the first factor that explained most of the variation was used to group study households into wealth tertiles.

Multinomial logistic regressions were employed to examine the relationship between famine exposure and Wealth index. In order to account for the effect of outstanding biologic and socio-demographic covariates three different regression models were used. In the first model, unadjusted coefficients of exposure on wealth index was computed, while model two and three were adjusted for biologic and demographic factors respectively. Hosmer-Lemeshow χ2 greater than 0.05 and maximum standard errors (SE) greater than 2 were used to check model fitness and multi-collinearity, respectively. Potential effect modification was assessed by interaction terms. The results were expressed as adjusted odds ratio and 95%confidence interval.

## Results

### Sociodemographic characteristics

A total of 968 participants were enrolled to the study. Of these three hundred thirty-three (34.4%), 302 (31.2%) and 333 (34.4%) were exposed in utero, postnatal and unexposed, respectively. The mean (± SD) ages for in utero, postnatal and unexposed groups were 35.05 (± 0.87), 37.63 (± 0.48) and 31.19 (± 0.66), respectively. One-hundred-ninety-seven (58.9%) of the participants were females exposed to famine in utero. Two- hundred-sixty-six (79.8%) of the participants were rural residents exposed to famine in utero. One-hundred-twenty-five (39.6%) of them were illiterate among in utero exposed groups (Table 1).

**Table 1:** Socio-demographic characteristics of the study participants according to Ethiopian famine exposure status, Wollo, Ethiopia (*N* = 968)

Variables	In Utero exposed (*n* = 333)	Postnatal exposed (*n* = 302)	Unexposed (*n* = 33)	*P-* value
Age, (years), mean ± SD	35.05 ± 0.87	37.63 ± 0.48	31.19 ± 0.66	< 0.001*
Sex, n (%)				
Female	197 (58.9%)	170 (60.2%)	152 (29.3%)	0.001*
Male	136 (41.1%)	132 (39.8%)	181 (51.7%)	
Residence, n (%)				
Urban	67 (20.1%)	45 (19.7%)	50 (15.2%)	0.124
Rural	266 (79.8%)	257 (80.3%)	283 (84.8%)	
Educational status, N (%)				
Illiterate	125 (39.6%)	123 (47.3%)	75 (24.8%)	< 0.001*
Primary school	85 (24.7%)	81 (24.1%)	62 (18.8%)	
Secondary school	72 (20.7%)	55 (16.3%)	115 (33.2%)	
Secondary and above	51 (14.9%)	43 (12.3%)	81(23.2%)	
Wealth index, N (%)				
Low	73 (21.8%)	100 (30.9%)	64 (20.0%)	0.001*
Medium	52 (15.8%)	42 (13.7%)	55 (16.6%)	
High	208 (62.4%)	160 (55.4%)	214 (63.4%)	
Marital status, N (%)				
Single	51 (15.5%)	35 (16.9%)	101 (29.2%)	< 0.001*
Married	222 (66.1%)	221 (68.2%	209 (62.8%)	
Divorced/Widowed	60 (18.4%)	46 (14.8%)	23 (8.0%)	
Occupation, N (%)				
Employed	89 (15.5%)	74 (15.5%)	112 (15.5%)	< 0.001*
Farmer	173 (15.5%)	152 (15.5%)	123 (15.5%)	
Merchant	54 (15.5%)	35 (15.5%)	64 (15.5%)	
Others**	17 (15.5%)	41 (15.5%)	34 (15.5%)	
Family Size	3.90 ± 1.60	3.80 ± 2.10	3.50 ± 1.40	0.18
Height, (cm), mean ± SD	163.30 ± 8.31	162.60 ± 7.60	163.38 ± 7.80	< 0.001*

*P*-value—t-tests for continuous variables or χ2-test for categorical variables,* Statistical significance, **students, daily laborer

### Association of early life famine exposure and economic achievent

Unadjusted coefficients of covariates on wealth tertiles are presented in Table 2. Accordingly, urban residents and female sex were found to have less odds to fall in low wealth tertile (*P* <  0.05). On the other hand, in all forms of famine exposure, participants who can’t ‘read or write and primary education are more than thrice and twice at risk of assuming lowest wealth tertile and middle wealth index respectively (*P* <  0.05). Moreover, participants with one unit higher MoCA score are 4–5% less likely fall in lowest wealth tertile (*P* <  0.05).

**Table 2:** Association of prenatal famine exposure and economic achievement in adults (unadjusted model), Multinomial logistic regression analysis, Wollo, Ethiopia (*n =* 968)

Variables	Wealth index tertiles	In utero exposed		Postnatal exposed		Unexposed
		Exp (β)	95%CI	Exp (β)	95%CI	Ref.
Female	Low	0.58*	0.38,0.88	0.99	0.69,1.40	Ref.
	Medium	0.70	0.42,1.17	0.75	0.49,1.14	Ref.
Urban	Low	0.63*	0.45,0.87	0.64	0.45,0.91	Ref.
	Medium	1.00	0.66,1.53	0.89	0.58,1.38	Ref.
Can’t ‘read or write	Low	3.37*	2.06,5.51	3.19*	1.80,5.66	Ref.
Primary education	Low	2.22*	1.31,3.76	2.49*	1.33,4.68	Ref.
Secondary education	Low	1.37	0.80,2.37	1.81	0.96,3.40	Ref.
Can’t ‘read or write	Medium	1.46	0.84,2.51	1.68	0.84,3.38	Ref.
Primary education	Medium	1.51	0.86,2.66	2.33*	1.13,4.80	Ref.
Secondary education	Medium	1.36	0.78,2.37	2.36*	1.18,4.75	Ref.
Age**	Low	1.05	1.02,1.09	1.04	1.01,1.07	Ref.
	Medium	1.00	0.97,1.03	0.99	0.96,1.03	Ref.
Family size**	Low	1.02	0.98,1.07	1.04	0.99,1.10	Ref.
	Medium	0.96	0.91,1.01	0.94	0.88,1.00	Ref.
MoCA Score**	Low	0.97*	0.96,0.99	0.98	0.97,0.99	Ref.
	Medium	0.99	0.97,1.00	1.02	0.99,1.01	Ref.
Height**	Low	1.00	0.98,1.01	0.99	0.98,1.04	Ref.
	Medium	1.00	0.97,1.01	1.00	0.98,1.03	Ref.

High wealth index, Male gender, rural residence, tertiary and above education were the references

*Statistically significant at *P* < 0.05, **Results for these variables are presented as beta coefficients, Ref—Reference

To explore the effect of the famine exposure on wealth, in unadjusted model (Model 1), compared to unexposed cohorts, in utero famine exposed cohort are nearly twice more likely to fall in lowest wealth tertile (OR: 1.93, 95%CI: 1.40, 2.64). Similarly, postnatal famine exposed cohorts are more more likely twice to fall in lowest wealth tertile (OR: 2.12, 95%CI: 1.45, 3.08). In model 2, adjusted for biologic variable (height, sex and age) these associations became non-significant for all types of exposures (*P* > 0.05). In model three, further adjustment was made for covariates including education, residence, family size and cognitive function and the association between the exposure and wealth remained non-significant (*P* > 0.5). Instead, in model three, educational status appeared significantly associated with wealth, where those who can’t read or write among in utero and postnatal exposed were three times more likely to fall in low wealth index category than those who had tertiary and above level of education (OR = 3.00 95% CI: 1.74, 5.18) and (OR = 2.92, 95% CI: 1.48, 5.76), respectively. Likewise, those with primary education among in uero and postnatal famine exposed cohorts were twice more likely to fall in the low wealth index than compared to those tertiary and above level of education (OR = 2.04 95% CI: (1.18, 3.54) and (OR = 2.17 95% CI: 1.12, 4.22), respectively (Table 3).

**Table 3:** Association of prenatal famine exposure and economic achievement in adults (adjusted model), Multinomial logistic regression analysis, Wollo, Ethiopia

Models	Wealth index	Variables	In utero exposed		Postnatal exposed		Unexposed
			Exp (β)	95%CI	Exp(β)	95%CI	Ref.
*Model 1*	Low	Exposed	1.93*	1.40, 2.64	2.12*	1.45, 3.08	Ref.
	Medium	Exposed	1.04	0.69,1.55	1.01	0.64, 1.59	Ref.
*Model 2*	Low	Age**	1.04	0.95, 1.14	0.88	0.63, 1.22	Ref.
		Height**	1.00	0.98, 1.02	0.98	0.96, 1.00	Ref.
		Female	1.00	0.73, 1.36	0.92	0.63, 1.34	Ref.
		Exposed	1.65	0.99, 2.75	4.97	0.59, 42.09	Ref.
	Medium	Age**	0.99	0.89, 1.09	0.92	0.63, 1.35	Ref.
		Height**	1.00	0.98, 1.02	1.00	0.98, 1.03	Ref.
		Female	0.95	0.66, 1.37	0.72	0.46, 1.14	Ref.
		Exposed	1.1	0.59, 2.03	1.75	0.15, 20.86	Ref.
*Model 3*	Low	Age**	0.99	0.90, 1.09	0.86	0.62, 1.21	Ref.
		Height**	1.00	0.98, 1.02	0.98	0.96, 1.00	Ref.
		Family size**	1.02	0.93, 1.11	1.00	0.90, 1.11	Ref.
		MoCA Score**	0.98	0.95, 1.01	1.00	0.96,1.04	Ref.
		Female	0.78	0.56, 1.08	0.73	0.48, 1.09	Ref.
		Urban residence	1.26	0.84, 1.89	1.15	0.67, 1.99	Ref.
		Can’t read or write	3.00*	1.74, 5.18	2.92*	1.48, 5.76	Ref.
		Primary education	2.04*	1.18, 3.54	2.17*	1.12, 4.22	Ref.
		Secondary education	1.39	0.8, 2.41	1.9	0.98, 3.61	Ref.
		Exposed	1.77	1.04, 3.00	4.74	0.53, 42.28	Ref.
	Medium	Age**	0.99	0.89, 1.09	0.97	0.66, 1.42	Ref.
		Height**	1.00	0.98, 1.02	1.00	0.98, 1.03	Ref.
		Family size**	0.91	0.81, 1.02	0.82	0.70, 0.95	Ref.
		MoCA Score**	0.98	0.95, 1.02	0.98	0.94, 1.03	Ref.
		Females	0.9	0.61, 1.31	0.69	0.42, 1.12	Ref.
		Urban residence	1.12	0.67, 1.85	1.67	0.89, 3.15	Ref.
		Can’t ‘read or write	1.41	0.77, 2.61	1.86	0.82, 4.18	Ref.
		Primary education	1.52	0.84, 2.75	2.24*	1.04, 4.81	Ref.
		Secondary education	1.34	0.77, 2.33	2.33*	1.15, 4.72	Ref.
		Exposed	1.05	0.56, 1.96	1.2	0.1, 14.99	Ref.

High wealth index, Male gender, rural residence, tertiary and above education were the references for

*Statistically significant at *P* < 0.05, **Results for these variables are presented as beta coefficients

## Discussion

The present study is conducted in the midst of limited evidence on the impact of childhood famine exposure on adulthood economic achievement. In the crude analysis, early childhood and postnatal famine exposure were found to have an inverse association with wealth achievement in adulthood, which are somehow in line with the findings of the Chinese famine study and Zimbabwe drought survivors which reported lower income and lifetime earning respectively [11, 24]. Taking interventional studies as surrogate, to complement the lack of similar studies for comparison, a study in Guatemala reported lower wage achievement among adults who didn’t receive supplementary feeding during childhood [34]. In the same approach, if low birth weight is considered as the proxy effect of famine, the findings related to adulthood economic outcomes such as employment rate, income, and likelihood of receiving social benefits are inconsistent [35–38]. However, these comparisons are in general generic and affected by observed as well unobserved covariates, thus difficult to generate any conclusions.

More importantly, the association between famine exposure and wealth fall insignificant when analyzed adjusted for biologic as well as sociodemographic covariates. Instead, education appears to be a significant independent factor to determine one’s economic achievement in the studied cohort. Notably, hierarchical differences in wealth index were also observed across education quartiles. The higher the school achievement, the lower the odds of falling in lowest wealth tertile was observed. Childhood famine exposed cohorts in the lowest education achievement were three times more likely to fall in lowest wealth tertile, while the corresponding odd for primary education and secondary education were decreasing, 2 and 1.39 respectively, as compared to tertiary and above educated. Similarly, among postnatal famine survivors, those who can’t read or write were nearly four times more likely to fall in lowest wealth tertile, while the corresponding odds for primary education and secondary education achiever were 2 and 1.39. This phenomenon peculiarly indicates the strong dose response relationship of education and wealth in the setting as best described the World Bank report [39].

Likewise, the crude effects of residence and cognitive assessment have shown associated with wealth achievement. However, adjusted for education, famine exposure and other biologic variables, all turned to be insignificant (*P* > 0.5). This occurrence reflects the power of education could have to modify the effect of biologic as well as demographic covariates over economic achievements. There exist many reports that justify the economic returns of schooling [39–41]. For example, in Central America, one additional year of schooling is associated with 12–14% increased lifetime earnings and much the same effect were estimated in Brazil [40, 41]. Contrary to what have been reported by most reviews, adult height has not been positively associated with wealth, after adjustment for education [42–44].

The present study provides empirical evidence on the contribution of education to modify the impact childhood famine exposure on future economic achievement by controlloing unobserved confounding effect of biologic as well as demographic factors. The implication of these findings should be interpreted in the view of the prevalent under nutrition in low income countries, which could have close or similar consequence. However, there remained certain limitation in our study, which could encumber the interpretation of the findings; first, it should note that survivors are naturally different likely introduce selection bias. Second; severity of exposure to famine at the individual level was not considered. Thirdly, early childhood experiences, parent–child bond and maternal factors during early life was not considered. Lastly, there still can be unknown confounders which can determine wealth achievement and yet not accounted in the present study.

In conclusion, the possible impact of early childhood famine exposure on future economic achievements during adulthood could be modified through better education. Our findings justify the need of expanding education in hunger spots in general and in old famine settings in particular.

## Supplementary Information

**Additional file 1.** Window of exposure to the 1983-1985 Ethiopian Great Famine cohorts, North Wollo Zone, 2019.

**Additional file 2: Figure 1.** Flow diagram representing sample recruitment.

## Data Availability

The data supporting the conclusions of this article is included within the article and its Additional file.

## Abbreviations

CI: Confidence interval
OR: Odds ratio
PCA: Principal Component Analysis
MOCA: Montreal Cognitive Assessment
SD: Standard Deviation
