# Editorial expression of concern: education can modify the long term impact of early childhood famine exposure on adulthood economic achievement: a historical cohort study among the survivors of the great Ethiopian famine 1983–85

**DOI:** 10.1186/s13690-026-01902-6

**Published:** 2026-03-31

**Authors:** Kalkidan Hassen Abate, Misra Abdullahi, Fedlu Abdulhay, Getachew Arage, Mohammed Mecha, Mohammed Yenuss, Habtamu Hassen, Tefera Belachew

**Affiliations:** 1 Department of Nutrition and Dietetics, Institute of Health, Jimma University, Jimma, Ethiopia; 2 Department of Population and Family Heath, Institute of Health, Jimma University, Jimma, Ethiopia; 3 Department of Obstetrics and Gynecology, Faculty of Medical Sciences, Jimma University, Jimma, Ethiopia; 4 Department of Nutrition and Dietetics, College of Health Sciences, DebreTabor University, Debre Tabor, Ethiopia; 5 Department of Internal Medicine, Faculty of Medical Sciences, Jimma University, Jimma, Ethiopia; 6 Department of Environmental Health Science, College of Health and Medical Sciences, Wollo University, Dessie, Ethiopia

**Editorial Expression of Concern: Arch Public Health 79, 67 (2021)**

**https://doi.org/10.1186/s13690-021-00564-w**

The Editors-in-Chief are issuing an editorial expression of concern to alert readers that the listed articles have been published by the same group of authors within a very close time frame contain similarities in text, indicating salami publications.

1. Arage G, Belachew T, Abera M, Abdulhay F, Abdulahi M, Abate KH. Women’s Experiences of the 1983-85 Ethiopian Great Famine: A Qualitative Study in Kobo Town, North Wollo Zone, Ethiopia. preprint (version1). 2020. Women’s Experiences of the 1983-85 Ethiopian Great Famine: A Qualitative Study in Kobo Town, North Wollo Zone, Ethiopia
2. Arage G, Belachew T, Hassen H, et al. Effects of prenatal exposure to the 1983–1985 Ethiopian great famine on the metabolic syndrome in adults: a historical cohort study. British Journal of Nutrition. 2020;124(10):1052-1060. 10.1017/S0007114520002123
3. Arage G, Belachew T, Abera M, et al. Consequences of early life exposure to the 1983–1985 Ethiopian Great Famine on cognitive function in adults: a historical cohort study. BMJ Open 2020;10:e038977. 10.1136/bmjopen-2020-038977
4. Arage, G., Belachew, T., Hajmahmud, K. et al. Impact of early life famine exposure on adulthood anthropometry among survivors of the 1983–1985 Ethiopian Great famine: a historical cohort study. BMC Public Health 21, 94 (2021). 10.1186/s12889-020-09982-x
5. Abate, K.H., Abdulahi, M., Abdulhay, F. et al. Consequences of exposure to prenatal famine on estimated glomerular filtration rate and risk of chronic kidney disease among survivors of the great Ethiopian famine (1983–85): a historical cohort study. Nutr J 20, 19 (2021). 10.1186/s12937-021-00675-8
6. Arage, G., Belachew, T., & Abate, K. H. (2022). Women's experiences related to the ‘great famine’ in Ethiopia: A qualitative study. International Journal of Disaster Risk Reduction, 82, 103329

The authors did not respond to correspondence from the Publisher about this Editorial Expression of Concern.
