# Supplementary material for: Education can modify the long term impact of early childhood famine exposure on adulthood economic achievement: a historical cohort study among the survivors of the great Ethiopian famine 1983–85
Source: Arch Public Health. 2021 May 4;79:67. doi: 10.1186/s13690-021-00564-w (PMC8097899; doi:10.1186/s13690-021-00564-w)
Supplement: Supplementary file 1 — Additional file 1. Window of exposure to the 1983-1985 Ethiopian Great Famine cohorts, North Wollo Zone, 2019. [file 13690_2021_564_MOESM1_ESM.docx]

Window of exposure to the 1983-1985 Ethiopian Great Famine cohorts, North Wollo Zone, 2019

| **Birth date of participants (dd/mm/yyy)** | **Exposure to the famine August 1983, August 1985** | **Age of exposure to famine** | **Age at recruitment** |
| --- | --- | --- | --- |
| 08/August/1983-30/August /1985 | In utero exposed | born or conceived during the famine | 34-36 |
| 08/September/1986 30/August/1987 | Transition (Washout period) | One years after the famine | 33 |
| 08/September/1981-30/August/1983 | Postnatal exposed | 0 – 2 years old | 37-38 |
| 08/September/1987-08/October/1988 | unexposed (control group) | Two years old after the famine | 30-32 |
