# Supplementary material for: Education can modify the long term impact of early childhood famine exposure on adulthood economic achievement: a historical cohort study among the survivors of the great Ethiopian famine 1983–85
Source: Arch Public Health. 2021 May 4;79:67. doi: 10.1186/s13690-021-00564-w (PMC8097899; doi:10.1186/s13690-021-00564-w)
Supplement: Supplementary file 2 — Additional file 2: Figure 1. Flow diagram representing sample recruitment. [file 13690_2021_564_MOESM2_ESM.docx]

Registration was carried out to prepared sampling frame

Total participants (n = 997)

Proportional allocation to selected kebeles then participants were selected by simple random sampling methods

Exclusion

Missing data (n= 29)

Participants included in the study (n= 968)

1. In Utero exposed (n = 333)
2. Postnatal exposed (n = 302)
3. Unexposed group **(**n = 333)

**Figure** **1**. Flow diagram representing sample recruitment.
